# Identification of Genes Differentially Expressed between Resistant and Susceptible Tomato Lines during Time-Course Interactions with *Xanthomonas perforans* Race T3

**DOI:** 10.1371/journal.pone.0093476

**Published:** 2014-03-31

**Authors:** Heshan Du, Wenhui Li, Yuqing Wang, Wencai Yang

**Affiliations:** Beijing Key Laboratory of Growth and Developmental Regulation for Protected Vegetable Crops, Department of Vegetable Science, China Agricultural University, Beijing, China; Key Laboratory of Horticultural Plant Biology (MOE), China

## Abstract

Bacterial spot caused by several *Xanthomonas* sp. is one of the most devastating diseases in tomato (*Solanum lycopersicum* L.). The genetics of hypersensitive resistance to *X. perforans* race T3 has been intensively investigated and regulatory genes during the infection of race T3 have been identified through transcriptional profiling. However, no work on isolating regulatory genes for field resistance has been reported. In this study, cDNA-amplified fragment length polymorphism technique was used to identify differentially expressed transcripts between resistant tomato accession PI 114490 and susceptible variety OH 88119 at 3, 4 and 5 days post-inoculation of the pathogen. Using 256 selective primer combinations, a total of 79 differentially expressed transcript-derived fragments (TDFs) representing 71 genes were obtained. Of which, 60 were up-regulated and 4 were down-regulated in both tomato lines, 4 were uniquely up-regulated and 2 were uniquely down-regulated in PI 114490, and 1 was specifically up-regulated in OH 88119. The expression patterns of 19 representative TDFs were further confirmed by semi-quantitative and/or quantitative real time RT-PCR. These results suggested that the two tomato lines activated partly similar defensive mechanism in response to race T3 infection. The data obtained here will provide some fundamental information for elucidating the molecular mechanism of response to race T3 infection in tomato plants with field resistance.

## Introduction

Bacterial spot severely affects tomato (*Solanum lycopersicum* L.) fruit production and quality in both open field and protected area [1]. The causal agent is a complex of at least four species of *Xanthomonas* (*X. euvescatoria*, *X. vescatoria*, *X. perforans*, and *X. gardneri*) with five races designated T1 through T5 based on their virulence on tomato differential genotypes [2]-[4]. Due to the existence of multiple pathogen species and races, marginal efficacy of commonly applied chemicals, development of resistance to these chemicals in bacterial populations, and a lack of available disease resistance traits in commercial cultivars, control of the disease has not been effective once epidemics start [5], [6]. Exploiting host resistance gene(s) combined with important defense response genes for developing cultivars with durable resistance is considered as an effective approach to manage the disease.

The resistance to race T3 of bacterial spot in tomato can be either qualitatively or quantitatively inherited. Two *S. pimpinellifolium* accessions PI 128216 and LA 1589 as well as one unimproved tomato breeding line Hawaii 7981 show both hypersensitive response (HR) and field resistance to race T3. The HR is conditioned by single dominant genes of *Xv3* in Hawaii 7981 [7], *Rx4* in PI 128216 [8], and *Rx_LA1589_* in LA 1589 [9]. These three genes have been mapped to the same region on chromosome 11 and might be the same gene or allelic genes [1]. Field resistance to race T3 in these lines shows partial or incomplete dominance, requiring interactions between *Xv3* and some modifiers in Hawaii 7981 [10], or depending on gene dosage and genetic backgrounds in PI 128216 [11].

The *S. lycopersicum* var. *cerasiforme* accession PI 114490 has been considered as a durable source for resistance to bacterial spot due to its high level of field resistance to four races T1-T4 [5]. Classical genetic analysis of resistance to race T2 using F_2_ and inbred backcross populations derived from PI 114490 suggests that genetic control is conferred by a minimum of two loci [12], and resistance to races T3 and T4 is conditioned by at least four quantitative trait loci (QTL) [13], [14]. A couple of QTLs are common for resistance to races T2, T3, and T4. Although there is no or few lesions on PI 114490, the bacterial population of race T3 in its leaves is not significantly different from that in susceptible variety OH 88119 [15]. In addition, PI 114490 does not show HR to the pathogen of bacterial spot [1]. Therefore, the mechanism of resistance in PI 114490 seems complicated and different from that in those lines with HR to the pathogen.

Despite extensive efforts have been made on understanding the genetic basis of resistance, little is known about the defense regulation and mechanism underlying inducible response to race T3 of bacterial spot in tomato. To date, a total of 426 genes differentially expressed during the time-course of HR to race T3 in Hawaii 7981[16] and 1345 genes triggered by recognition of the *Xanthomonas* type III effector AvrXv3 [17] have been identified. These genes have been predicted to participate in a complex molecular network of regulation including components of defense responses, stress transcriptional regulation factors, signal transduction components, and regulators of primary and secondary metabolisms. Furthermore, several genes involved in the disease resistance process have been isolated through this approach [18], [19]. However, these studies are based on hypersensitive response to the pathogen of bacterial spot in tomato plants and may not cover all genes during the process of field resistance.

The complementary DNA-amplified fragment length polymorphism (cDNA-AFLP) is a powerful method to obtain a wide collection of differentially expressed transcript profiles even if rarely expressed during the process of response to abiotic or biotic stresses [20]–[22], enabling discovery of novel genes without any prior knowledge of gene sequences [23]. Moreover, compared with hybridization-based approaches including DNA chips and microarrays, cDNA-AFLP has a relatively low startup cost and enables distinguishing expression patterns of highly homologous gene family members [24]. The cDNA-AFLP technique has been successfully employed to identify differentially expressed genes in various plant-pathogen systems [22], [23], [25]–[27]. In this study, we isolate 71 genes differentially expressed in resistant line PI 114490 and susceptible line OH 88119 during time-course of response to bacterial spot race T3 using the cDNA-AFLP technique. The expression patterns of 19 genes are validated using semi-quantitative RT-PCR and/or quantitative real time RT-PCR (qRT-PCR). The data obtained here will provide some basic information for understanding the molecular mechanism of response to bacterial spot race T3 infection in tomato plants with field resistance.

## Materials and Methods

### Plant materials

Two tomato lines, the *S. lycopersicum* var. *cerasiforme* accession PI 114490 with field resistance and a variety OH 88119 susceptible to *X. perforans* race T3 [14], were used in this study. All seeds were surface sterilized with 4% NaClO for 5 min and germinated in 288 Square Plug Trays (Taizhou Longji Gardening Materials Co., Ltd, Zhejiang, China) in a sterilized mixture of peat and vermiculite (3:1). One-month old seedlings were transplanted into 10 cm (diameter) x 8 cm (height) pots filled with the same sterilized peat : vermiculite mixture and placed in a growth room with 28/25°C day/night cycle and 14 h photoperiod. Water and fertilizer were provided as needed.

### Inoculum preparation and inoculation

*X. perforans* race T3 strain *Xv829* was grown on yeast, dextrose, and calcium carbonate (YDC) agar medium [28] at 28°C for 48 to 72 h. Bacterial cells were collected and resuspended to A_600_  =  0.2 (approximately 3×10^8^ colony forming units per ml) using sterile solution containing 10 mM MgSO_4_·7H_2_O and 0.025% (v/v) Silwet L77. About five to six- week old seedlings were spray-inoculated with bacterial suspensions, whereas control plants (mock-treatment) were sprayed with the same sterile solution. Leaf samples were collected from the infected and mock-treated plants at 3, 4, and 5 days post-inoculation (DPI) for RNA isolation.

### RNA isolation and cDNA synthesis

Total RNA was isolated from both infected and mock-treated leaves using Trizol reagent following the manufacturer’s instructions (Invitrogen, CA, USA). The RNA integrity was monitored by 1.2% (w/v) agarose gel electrophoresis and the concentration was determined using a Nanodrop 2000 Spectrophotometer (Thermo Fisher Scientific, DE, USA). mRNA was isolated following the protocol of polyA Tract mRNA isolation system kit (Promega, WI, USA), and used as the template for double-stranded cDNA synthesis using M-MLV reverse transcriptase (TaKaRa, Dalian, Liaoning, China). The double-stranded cDNAs were purified using phenol/chloroform extraction and ethanol precipitation, and then stored at -20°C.

### cDNA-AFLP analysis

The template for cDNA-AFLP was prepared using the method described in Bachem et al. [29]. Approximately 100 ng cDNA from each sample was first digested with restriction enzyme *EcoR* I at 37°C for 8 h, and then with *Mse* I at 65°C for 8 h. The double-stranded adapters were ligated to the digested products using T4 DNA ligase (Promega) at 16°C overnight. After ligation of the adapters, 1 μl diluted ligation mixture (1∶10) was used as the template for pre-amplification in a 20 μl reaction mixture containing 10 μl *Taq10* 2×Master Mix (Ausable Biotechnology, Beijing, China), 8 μl ddH_2_O, and 0.5 μl (10 μM) pre-amp primers. The PCR thermal cycling parameters were as follows: 94°C for 3 min, 26 cycles of 94°C for 30 s, 56°C for 1 min, and 72°C for 1min, and a final extension at 72°C for 5 min. The pre-amplified products were diluted (1:50) with ddH_2_O for selective amplification using 256 AFLP selective primer combinations under the following conditions: 94°C for 3 min, 12 cycles of 94°C for 30 s, 65°C for 30 s (decrease by 0.7 each cycle), and 72°C for 1 min, then by 23 cycles at 94°C for 30 s, 56°C for 30 s, and 72°C for 1min, and an extra extension at 72°C for 5 min for the last reaction. Following the selective amplification, 10 μl of each PCR product was mixed with 2 μl of formamide stop/loading buffer (95% formamide, 20 mM EDTA, pH 8.0, and bromophenol blue), denatured at 98°C for 10 min, and then chilled immediately on ice. The products were separated on 7% denaturing polyacrylamide gel at a constant power (95W) for 4.5 h using 20 cm×45cm gel apparatus (Model: JY-CX1B, Beijing Junyi-Dongfang Electrophoresis Equipments Co., Ltd, Beijing, China ) and visualized using the silver-staining approach [30].

### Isolation and sequence analysis of differentially expressed transcript-derived fragments (TDFs)

AFLP fragments with persistent up- or down-regulated at 3, 4 and 5 DPI were excised from polyacrylamide gels with a clean razor blade, directly rinsed with 200 μl TE buffer (10 mM Tris and 1 mM EDTA, pH 8.0), and then incubated in 100 μl TE for 10 min using a boiling water bath. The fragments were re-amplified using the same conditions of selective amplification protocol described above with the corresponding primers. The resulting PCR products were separated on 1.5% agarose gel and purified using Agarose Gel purification Kit (Biomed Co., Beijing, China).

The purified PCR products were ligated into the pMD19-T vector (Takara), transformed into *E. coli* DH5α competent cells, and plated on Luria-Bertani (LB) agar medium containing 50 μg/ml ampicillin. The recombinant clones were verified by PCR, and at least two positive clones were sequenced at Sunbiotech Company (Beijing, China). Each sequence was searched against the International Tomato Annotation Group (ITAG) Release 2.3 Predicted CDS (SL2.40) in the Sol Genomics Network (SGN) (http://solgenomics.net) database using BLASTX program to obtain the function annotation. Gene ontology analysis was performed using GoPipe [31] through BLASTP against Swiss-Prot and TrEMBL database. The WEGO software (http://wego.genomics.org.cn) was used for gene ontology (GO) classification and plotting [32].

### RT-PCR and qRT-PCR analysis

To confirm the results of cDNA-AFLP analysis, 19 strongly differentially expressed TDFs were chosen for semi-quantitative RT-PCR and/or qRT-PCR analysis. Since the sequence lengths of TDFs were short, the sequences of corresponding CDS from SGN were used to design gene-specific primers. The specific primers for TDF792 were designed using the sequence of 3′UTR region obtained using the SMARTer RACE cDNA Amplification Kit (Clontech Laboratories Inc., CA, USA). The specificity of each primer pair was confirmed by analyzing PCR products on agarose gel and melting curve during real-time PCR.

RT-PCR was performed using the method described in Pei et al. [8], and the products were separated on 1.2% agarose gel, stained with ethidium bromide, and photographed using a GIAS-4400 Gel Documentation System (Beony Science and Technology Co., Beijing, China). qRT-PCR was conducted in a 20 μl reaction volume consisting of 10 μl SYBR-Green supermix (TaKaRa), 0.2 μl ROXII, 0.2 μl each of forward and reverse primers (10 μM), and 9.4 μl cDNA templates diluted (1:40) from the first strand cDNA solution prepared for cDNA-AFLP. Reactions were heated at 95°C for 3 min followed by 40 cycles of 95°C for 15 s, 57°C for 30 s, and 72°C for 15 s using the ABI 7500 machine (Applied Biosystems, CA, USA). All samples were performed in triplicate with the control sample as the internal reference. The relative expression of the target genes was calculated by normalizing Ct values in each sample against the *EF1-α* using comparative C-T method [33].

## Results

### Responses to *X. perforans* race T3 in OH 88119 and PI 114490

**Figure 1:**
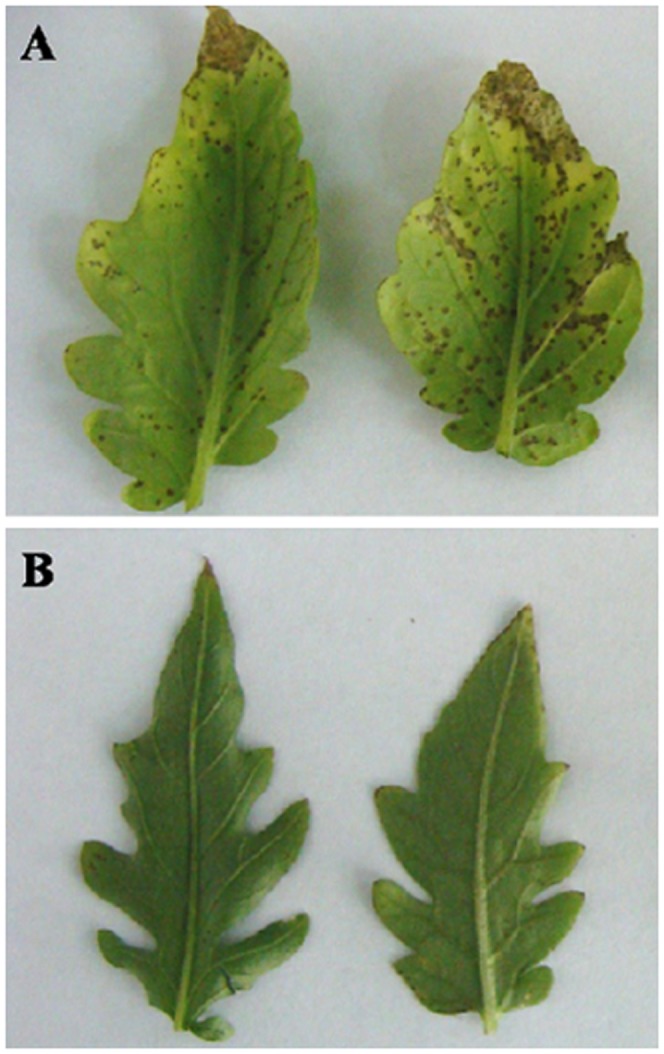
Foliar lesions on tomato plants after spray-inoculation with bacterial spot race T3 (*Xanthomonas perforans*) strain *Xv829*. A: Lesions on a leaf of the susceptible line OH88119. B: Lesion on a leaf of the resistant line PI 114490.

The development of disease on plants was monitored each day after inoculation. No symptom was observed on both PI 114490 and OH 88119 plants prior to 4 DPI. Small lesions with yellow haloes developed on the leaves of OH 88119 on 5 DPI, and subsequently expanded to form large necrotic areas throughout the inoculated leaves in the late stage of the disease (Fig. 1A). In contrast, PI 114490 always showed few and small lesions restricted to portions of the leaves (Fig. 1B). This was consistent with our previous observations [15].

Based on the progress of disease development, the time-course transcriptional profiling of PI 114490 and OH 88119 were conducted to identify differentially expressed genes during the process of race T3 infection. Infected and mock-treated leaves were harvested at 3, 4 and 5 DPI for RNA isolation and cDNA-AFLP analysis.

### Isolation and analysis of differentially expressed TDFs

**Figure 2:**
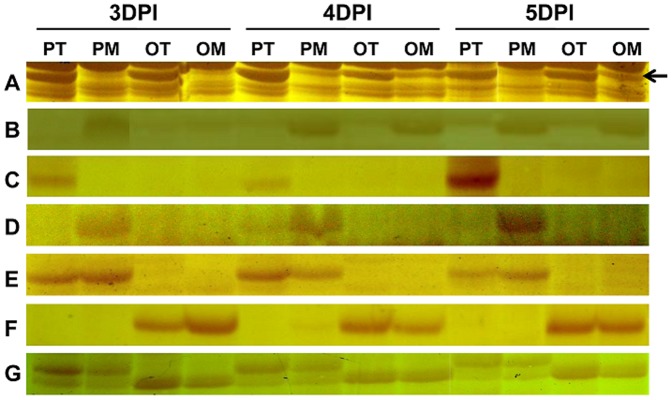
Enumerating main types of differentially expressed fragment during cDNA-AFLP analysis. PT: PI 114490 inoculated with T3. PM: PI 114490 mock-inoculated with the sterile solution containing 10 mM MgSO_4_·7H_2_O and 0.025% (v/v) Silwet L77. OT: OH 88119 inoculated with T3. OM: OH 88119 mock-inoculated with the sterile solution containing 10 mM MgSO_4_·7H_2_O and 0.025% (v/v) Silwet L77. A: Common persistent up-regulated TDF (indicated by an arrow) in both tomato lines. B: Common persistent down-regulated TDF in both tomato lines. C: Specific persistent up-regulated TDF in PI 114490. D: Specific persistent down-regulated TDF in PI 114490. E: Constitutive expressed only in PI 114490. F: Constitutive expressed only in OH 88119. G: Length polymorphism fragment between two tomato lines.

A total of about 6400 TDFs with sizes of 100 bp to 500 bp were amplified using 256 AFLP selective primer combinations. Various types of differentially expressed TDFs between PI 114490 and OH 88119 at three time-points (3, 4, and 5 DPI) were observed, which mainly included commonly and persistently up- or down-regulated in both lines (Fig. 2a,b), specifically up- or down-regulated in either PI 114490 or OH 88119 (Fig. 2c,d), constitutively expressed only in PI 114490 or OH 88119 (Fig. 2e-g). Considering the occurrence of fragments unique to PI 114490 or OH 88119 might be due to the mutation of restriction sites for *EcoR* I or *Mse* I, only TDFs commonly presented in both lines with persistently up- or down-regulated at three time-points were excised from the polyacrylamide gel. Therefore, 122 TDFs were recovered from the gels, and 108 were successfully sequenced. Due to inconsistency between sequences derived from two clones, 29 TDFs were discarded and the remaining 79 TDFs were retained for further analysis (Table 1). Among the 79 TDFs, 68 (86.1%) showed up-regulated and four showed down-regulated expression in both PI 114490 and OH 88119, four were specifically up-regulated and two were specifically down-regulated in PI 114490, and one displayed specifically up-regulated in OH 88119 (Table 1).

**Table 1:** Differentially expressed patterns of TDFs during inoculation with race T3 and their annotation detected in Sol Genomics Network database.

TDF No.	Primer combination^a^	Length (bp)^b^	Annotation^c^	Gene locus from ITAG2.3
**Up-regulated in both genotypes**				
773/349	AG-CA/GG-AA	157/305	Kunitz-type protease inhibitor	Solyc03g098780.1.1
263/670/790	TA-GC/AA-CT/TA-AG	282/102/129	Cysteine proteinase inhibitor	Solyc00g071180.2.1
741	CA-GC	305	Jasmonate ZIM domain 2	Solyc03g122190.2.1
721	CA-CT	321	1-aminocyclopropane-1-carboxylate oxidase	Solyc07g049530.2.1
30	TT-AA	208	Chitinase	Solyc10g055800.1.1
671	AT-GG	177	Malic enzyme	Solyc05g050120.2.1
720	GG-CT	334	Polyphenol oxidase	Solyc08g074680.2.1
656	CG-AA	382	N-acetyltransferase	Solyc08g068710.1.1
794	TT-CA	119	Homology to unknown gene	Solyc08g066600.2.1
300	AT-TG	95	Subtilisin-like protease	Solyc01g087850.2.1
108	TG-AT	180	Subtilisin-like protease	Solyc10g084320.1.1
13	TA-AA	287	Cathepsin B	Solyc02g069100.2.1
270	GC-TT	112	Peroxidase	Solyc03g006700.2.1
24	AC-AT	257	Lipase-like protein	Solyc04g010250.2.1
792	TT-GC	108	U-box domain-containing protein	Solyc11g068940.1.1
289	AT-AG	179	Peroxisomal membrane protein	Solyc01g091730.2.1
710	GA-CG	122	Serine carboxypeptidase S28 family protein	Solyc03g033620.2.1
297	AT-TG	129	Pheromone receptor-like protein	Solyc04g079350.1.1
673	AT-CA	125	ATP-binding cassette 1	Solyc04g015970.2.1
318	AC-TC	240	Phenylalanine ammonia-lyase	Solyc09g007920.2.1
308	AG-AT	205	Short-chain dehydrogenase/reductase protein	Solyc07g047800.2.1
22/12/791	TA-AA/TA-AA/TT-GC	246/296/180	heat shock protein 70	Solyc09g010630.2.1
317	AC-TC	277	Anthocyanidin synthase	Solyc10g076540.1.1
255	TA-GT	363	HhH-GPD family protein	Solyc11g007580.1.1
702	TG-CC	125	peroxidase	Solyc01g105070.2.1
626	CA-TG	190	Diacylglycerol O-acyltransferase	Solyc01g095960.2.1
701/624	TG-CG	228/455	Spermidine synthase 1	Solyc03g007240.2.1
745	CT-GC	291	Serine carboxypeptidase 1	Solyc06g083020.1.1
281	AA-AC	146	Cytochrome P450	Solyc09g098030.2.1
711	GT-GG	227	Cytochrome P450	Solyc04g079730.1.1
81	GT-GT	218	hydroxycinnamoyltransferase-like protein	Solyc11g071480.1.1
770	AG-GG	106	Glutamyl-tRNA (Gln) amidotransferase	Solyc11g071550.1.1
781	GC-CC	146	Wound-induced basic protein	Solyc06g083340.2.1
744	CA-CA	73	Jasmonate ZIM-domain protein 3	Solyc01g005440.2.1
87	GT-AT	251	AP2-like ethylene-responsive transcript factor.	Solyc11g072600.1.1
668	AA-CT	107	Calmodulin-binding protein	Solyc07g006830.2.1
260	AC-GG	173	Heat shock protein 90	Solyc12g015880.1.1
321	TT-GT	205	Heat shock protein DnaJ domain protein	Solyc02g062350.2.1
319	AC-TC	126	Heat shock protein 90	Solyc07g065840.2.1
657/662	CG-AA/CG-CA	149/140	TO54-2	Solyc06g024210.1.1
775	TG-CG	154	60S ribosomal protein	Solyc12g044720.1.1
655	CT-TC	111	Glycogen synthase	Solyc03g083090.2.1
348	GG-AA	199	Cell division protease FtsH homolog	Solyc02g032960.2.1
654	CT-TC	86	Receptor like kinase, RLK	Solyc10g081910.1.1
712/83	GT-CT/GT-GA	235/220	Alcohol dehydrogenase zinc-binding protein	Solyc12g010960.1.1
601	GC-TG	149	Protein phosphatase 2C containing protein	Solyc08g077150.2.1
600	GC-TG	141	Myb transcription factor	Solyc03g112390.2.1
776	TG-CA	229	Chalcone isomerase protein	Solyc02g067870.2.1
667	AA-CA	466	PAS	Solyc07g017740.2.1
61	TC-TA	214	Ycf2	Solyc01g007640.2.1
66	TC-TT	213	5&apos-bisphosphate nucleotidase-like protein	Solyc02g079250.2.1
11	TA-AA	385	Heme oxygenase 1	Solyc12g009470.1.1
746	CT-GC	291	Serine carboxypeptidase 1	Solyc06g083040.2.1
666	CG-TC	111	Ras-related protein Rab-25	Solyc09g098170.2.1
789	TT-GC	122	F-box/LRR-repeat protein 3	Solyc10g076290.1.1
771	AG-GC	105	Amino acid transporter	Solyc02g065680.2.1
777	TG-CA	236	Adenosine kinase	Solyc10g086190.1.1
606	GC-TC	183	CHP-rich zinc finger protein-like	Solyc01g073840.1.1
772	AG-CA	159	no hits found	
742	CA-GC	280	no hits found	
**Down-regulated in both genotypes**				
625	CA-TG	311	Os07g0175100 protein	Solyc07g040960.1.1
115	TG-AC	225	Unknown Protein	Solyc07g056640.1.1
52	TC-AC	254	Single-stranded DNA binding protein	Solyc10g086150.1.1
780	GC-CG	168	Unknown Protein	Solyc01g103110.2.1
**Specifically up-regulated in PI 114490**				
795	GA-GC	156	Cell division protease ftsH	Solyc03g007760.2.1
740	CG-CA	140	Proline rich protein	Solyc12g009650.1.1
747	CT-CA	212	no hits found	
785	AT-GC	280	no hits found	
**Specifically up-regulated in OH 88119**				
352	CA-AA	301	Myb transcription factor	Solyc09g011780.2.1
**Specifically down-regulated in PI 114490**				
262	TA-GC	185	expressed protein	Solyc09g010540.2.1
17	TA-AA	229	Heavy-metal-associated domain-containing protein	Solyc02g087150.2.1

a Two selective nucleotide at the 3′terminus of each primer.

b Multiple cDNA length indicates different TDFs with different sequences from the same gene.

c “No hits found”indicates that the sequence of TDF do not show any sequence similarity to known cDNA sequences in ITAG Release 2.3 Predicted CDS (SL2.40) database.

Based on the ITAG annotation, the 79 TDFs represented 71 genes (Table 1). Four TDFs (TDF742, TDF747, TDF772, and TDF785) did not show sequence similarity to any gene in the tomato predicted CDS database. However, searching these four TDFs in tomato WGS Chromosomes (SL2.40) sequences indicated that TDF742 and TDF785 were probably derived from 3′ non-coding sequences of Solyc00g009760.2.1 (Cytochrome P450 monooxygenase) and Solyc11g051200.1.1 (Cytochrome P450 like_TBP), respectively. TDF772 was probably derived from 5′ non-coding sequence of Solyc07g065890.2.1 (Uridine kinase). TDF747 had high sequence identity (97.6%) to SGN-E258658.

### Gene ontology enrichment analysis of differentially expressed genes

**Figure 3:**
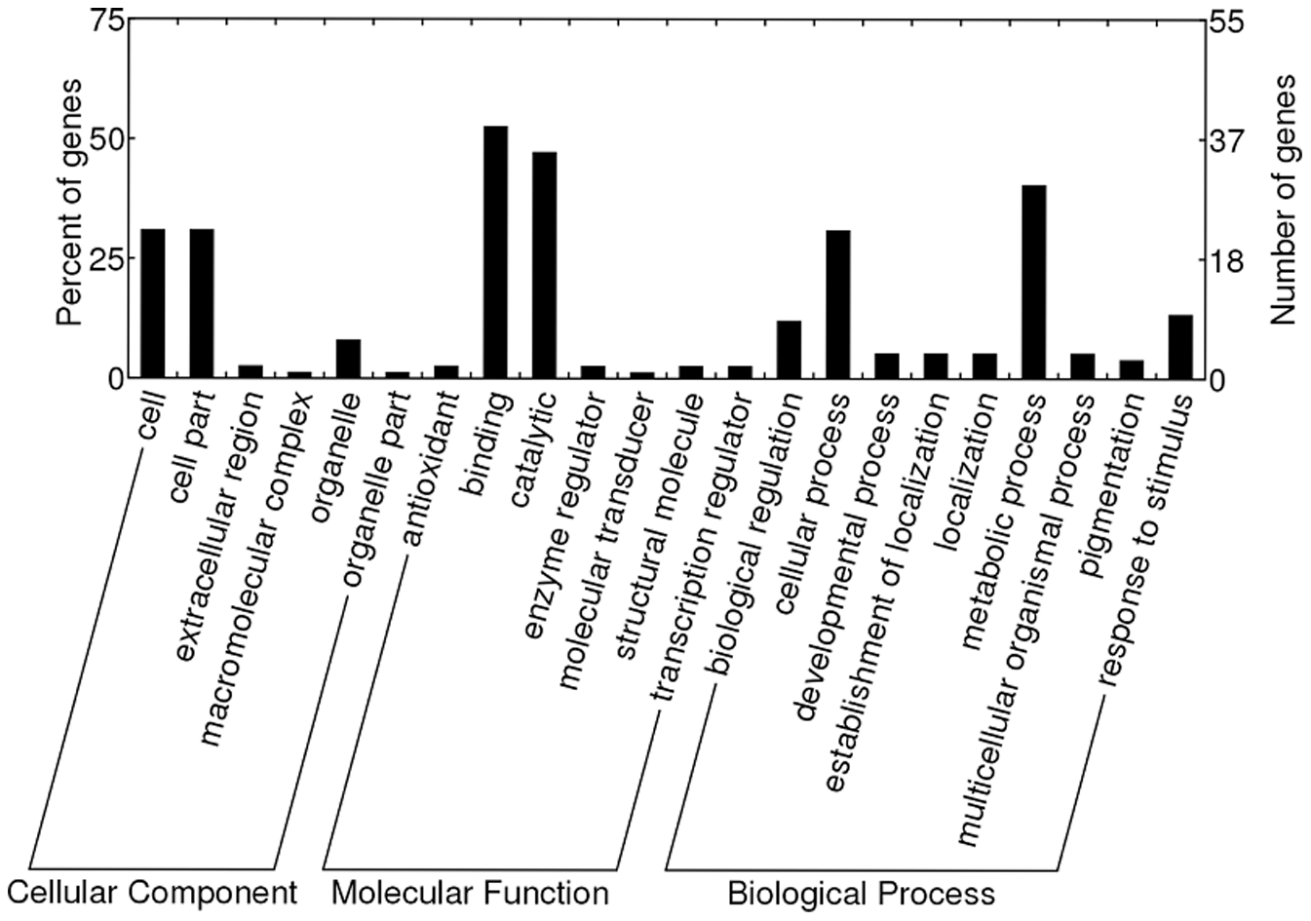
Gene classification based on Gene ontology (GO) enrichment for differentially expressed genes.

Based on the functional annotation using GoPipe [31], 61 of the 71 differentially expression genes had GO terms. Gene ontology enrichment analyses assigned the 61 genes to 22 functional groups in three main categories including cellular component, molecular function, and biological process (Fig. 3). The dominant subcategories for each main category were cell, binding, and metabolic process, respectively. In molecular function category, genes involved in catalytic activity also well represented, while the cellular component category mainly consisted of cell part. It was noteworthy that 11 differentially expressed genes involved in “response to stimulus” category (GO:0050896) within biological process. With the exception of TDF352 that was specifically up-regulated in OH 88119, the remaining 10 TDFs (30, 270, 702, 260, 318, 791, 352, 600, 87, and 255) were up-regulated in both PI 114490 and OH 88119. Functional annotation analysis revealed that some differentially expressed TDFs that were not classified into the ‘response to stimulus’ subcategory could also be induced during other biotic and abiotic stresses. The TDF781 without GO term showed approximately 91.5% of amino acid identity with PvPR4, one of the smallest wound-induced proteins that mRNA expression level increased 10-fold upon wounding over a period of 24 h in *Phaseolus vulgaris* [34]. Another TDF108 (Solyc10g084320.1.1, Subtilisin-like protease) not assigned to the ‘response to stimulus’ subcategory was significantly up-regulated in tomato plants with response to *Clavibacter michiganesis* subsp. *michiganesis* at 4 and 8 DPI [35].

### Validation of expression patterns for selected genes

To select a reliable reference gene for RT-PCR and qRT-PCR analysis, the expression of commonly used reference genes (*EF1-α*, 18S rRNA and *Ubi*) was analyzed with the same sample used for cDNA-AFLP analysis. When cDNA concentration of all the samples was adjusted to 0.4 μg/20 μl in qRT-PCR reaction system, the sample’s Ct values of *EF1-α* and 18S rRNA were at ranges of 20.3–20.9 and 16.2–16.9, respectively, while the expression profiles of *Ubi* varied among samples (data not shown). Combining the current results and internal reference genes used in literatures [36], [37], the *EF1-α* was used to normalize the amount of cDNA of all samples.

A total of 19 strongly modulated TDFs (Table 2) were used to validate the cDNA-AFLP results. Based on the functional annotation in literatures, majority of these TDFs were associated with massive defense response processes including jasmonic acid biosynthesis pathways (TDF711and TDF741), ethylene biosynthesis (TDF87 and TDF721), defense-related enzymes (TDF30, TDF657, TDF673 TDF702, TDF108, TDF701, TDF712, and TDF745), and cell death related (TDF792 and TDF781). The remaining (TDF625, TDF626, TDF666, TDF667, and TDF794) did not match any known defense response genes.

**Table 2:** Sequences of specific primer pairs used for RT- and qRT-PCR amplification.

TDF No.	Forward primer sequence (5′-3′)	Reverse primer sequence (5′-3′)
30	ATGACCCCTCAATCACCAAAG	TGTCACCAGGACTAACTCCAAGA
87	GCCGCAATCAAATGTAATG	TCCTGCTGGCTACCTTCAC
108	TCCATTGCTGCTGGTAGTCC	TCAGAAGTGACGGTCCCTTCT
625	AAAGGTCTCGTCAGGGTTCT	TGTAGCCAATCTCCAAATGC
626	TTGTGTTAGTTCCGTTGAGTATCG	CCCAAACAAGTTGAGGAAAAATT
657	CTCTTCCACTTTTCTACGGTCC	CCCAACTTCCTATAACTCTCCC
666	ACGTTTGTTGAATCGAGGG	CAGATGATGAGGGTGAGGAG
667	GATGTCTTCGTTTGGCATAT	TTTCGTGATGGAGTGGGATA
673	ATCGGCGGCAGAAATAAG	TTGAAATGTGGCGTCAGG
701	TCTCTCTCGGATTCCTTCTTCTTTT	CTTCCCATCCATAGTCCTCC
702	AGCATTATGTCCACAAAACGG	CTCAATCCAAGAAATCCCCTG
711	TTCATCGGAGAAGAAGGGGAG	GACCAGCAACAATCTTGAAACC
712	TTCATGGTCGTATTGCTGTG	GAGCACCAGGAGCACTTTCA
721	GGGACATTACAAGAAGTGCA	GCTTAGGACATGGTGGATAG
741	ACCTCCAGATTAAGCCAGAC	GCTTAGGACATGGTGGATAG
745	AAAAGGTATTGAGCTGGAGTA	ATAAGTCATCTGAAATGAGGC
781	CCTCTGTTCCTTGGGCTTCT	CCAACGACGATGATTTACGAC
792	CTTTTGACAAAACAATAGAATAG	ATTGTGATTTCCAACTTTCTA
794	AGGAAGAATGCGTCTAAAGTT	CAGTGATGATGGTGGAAAGG
EF1-α	TACTGGTGGTTTTGAAGCTG	AACTTCCTTCACGATTTCATCATA
18SrRNA	AAACGGCTACCACATCCAAG	CCTCCAATGGATCCTCGTTA
Ubi	GAAAACCCTAACGGGGAAG	GCCTCCAGCCTTGTTGTAAA

**Figure 4:**
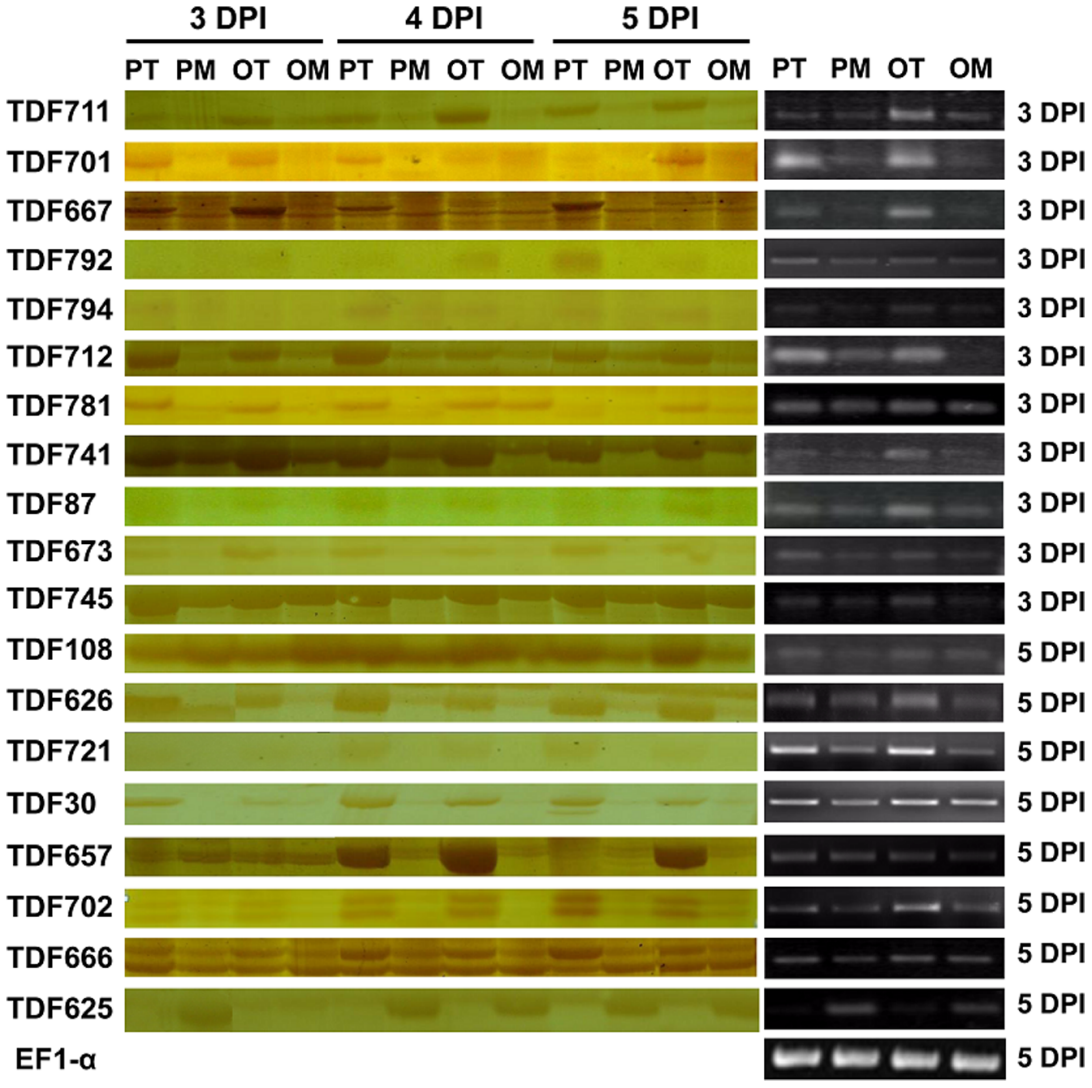
Images of cDNA-AFLP and Semi-quantitative RT-PCR analysis of 19 representative TDFs in leaves of tomato lines PI 114490 and OH 88119 at various time-points after inoculation of bacterial spot race T3 strain *Xv829*. PT: PI 114490 inoculated with T3. PM: PI 114490 mock-inoculated with the sterile solution containing 10 mM MgSO_4_·7H_2_O and 0.025% (v/v) Silwet L77. OT: OH 88119 inoculated with T3. OM: OH 88119 mock-inoculated with the sterile solution containing 10 mM MgSO_4_·7H_2_O and 0.025% (v/v) Silwet L77. The EF1-α was used as a constitutive control for the RT-PCR.

**Figure 5:**
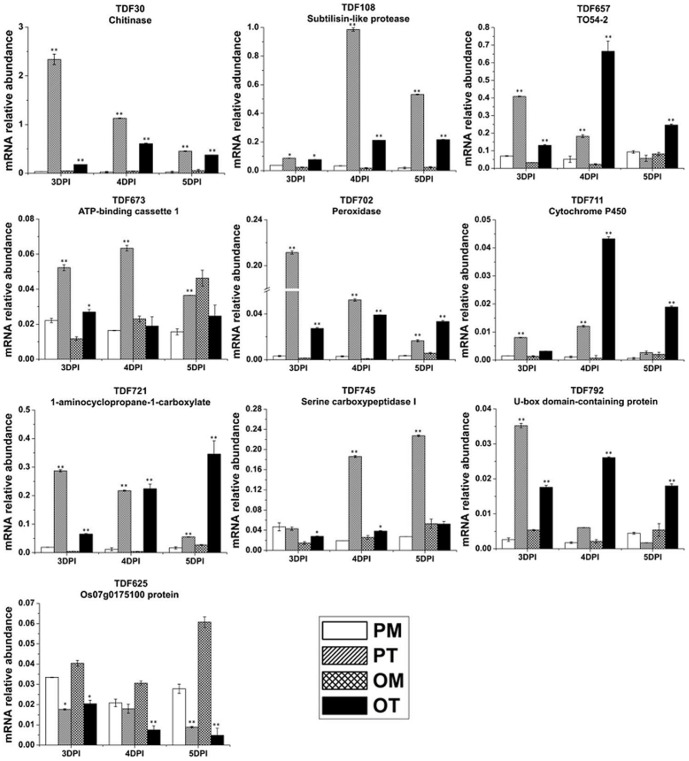
qRT-PCR analysis of 10 differentially expressed TDFs in tomato. PT: PI 114490 inoculated with T3. PM: PI 114490 mock-inoculated with the sterile solution containing 10 mM MgSO_4_·7H_2_O and 0.025% (v/v) Silwet L77. OT: OH 88119 inoculated with T3. OM: OH 88119 mock-inoculated with the sterile solution containing 10 mM MgSO_4_·7H_2_O and 0.025% (v/v) Silwet L77. Gene expression was determined relative to *EF1-α* transcript levels in the same samples. The data are means SD of three experimental replicates. The asterisk above the bars indicates statistically significant differences between the infected samples and corresponding mock treatment.

The first strand cDNA of 3 or 5 DPI was used for the RT-PCR validation (Fig. 4). Except that TDF625 was down-regulated, other 18 TDFs showed up-regulation in both PI 114490 and OH 88119 upon race T3 infection, which were consistent with cDNA-AFLP data. qRT-PCR was used to confirm the fold change of expression levels for 10 TDFs at 3, 4, and 5 DPI. It was very clear that TDF625 was down-regulated and the other nine TDFs were up-regulated at different time-points of infection in both PI 114490 and OH 88119, differing in their induction timing and the strength of expression patterns (Fig. 5). TDF30, DF702 and TDF721 displayed significantly high fold-induction in both tomato genotypes during three time-points of infection. At 3 DPI, the expressions of eight TDFs (30, 108, 702, 721, 711, 657, 673, and 792) with homologies to pathogenesis or defense-related genes were significantly induced to higher levels in resistant PI 114490 than susceptible OH88119. However, the up-regulated fold changes of TDF721, TDF711 and TDF657 were then reinforced significantly only in OH 88119 at 4 and 5 DPI. The expression level of TDF745 was significantly up-regulated in OH 88119 at 3 DPI, but fold change value was higher in PI 114490 at 4 and 5 DPI. These results were almost consistent with cDNA-AFLP analysis, indicating the reliability of cDNA-AFLP results.

## Discussion

The progresses on understanding genetics of host resistance [1] and the emergency of the pathogen genome sequencing [38], [39] have urged bacterial spot to be a suitable pathosystem for investigating the mechanism of tomato-*Xanthomonas* interactions. Although several loci conferring HR to races T1, T3, or T4 of bacterial spot have been mapped [1], [5], and a candidate gene *Rx4* conferring HR to race T3 in *S. pimpinellifolium* accession PI 128216 and genes responding to race T3 in tomato line Hawaii 7981 during the process of HR have been identified [8], [16], [17], none of these HR genes has been cloned. Therefore, the mechanism of resistance to bacterial spot in tomato remains unclear. Particularly, less work has been done on identification and characterization of genes during the disease process in tomato lines with only field resistance. Using the cDNA-AFLP approach, we here identified 79 TDFs differentially expressed during the disease process in resistant line PI 114490 and susceptible tomato line OH 88119. The expression patterns of some TDFs were further verified by RT-PCR and/or qRT-PCR, suggesting that these TDFs might contribute to host response to the pathogen of bacterial spot during the disease process in tomato. This will provide some fundamental information for investigating the mechanisms of resistance to bacterial spot in tomato.

The number (71) of differentially expressed genes identified in tomato lines PI 114490 and OH 88119 during the disease process was lower comparing to a total of 426 genes identified from tomato line Hawaii 7981 [16]. This could be due to several reasons. First, different techniques might contribute to the difference in numbers of genes identified in two studies. Suppression subtractive hybridization and microarray analysis were used in the previous study [16], while cDNA-AFLP technique was used in this study. Without parallel experiments using these techniques in one study, it was hard to tell the difference in terms of efficiency between two techniques. Second, different criteria might cause different numbers of genes identified in two studies. Gibly et al. [16] only used the resistant tomato line Hawaii 7981 to identify differentially expressed genes with RNAs isolated from pooled leaf tissues collected at various time-points after inoculation. Here we used two tomato lines including one resistance line PI 114490 and one susceptible line OH 88119, and only genes persistently up- or down-regulated at 3, 4, and 5 DPI in both lines were selected. This approach excluded a lot of genes with different expression patterns at three time-points in two tomato lines, which might result in loss of some genes involved in the disease process. Third, different responses to race T3 of tomato bacterial spot in two resistance lines and different inoculation method might also contribute to different numbers of differentially expressed genes identified in two studies. Gibly et al. [16] used infiltration inoculation to incite HR in Hawaii 7981 and we used spray inoculation to evaluate field resistance in PI 114490. Plants with HR should develop localized cell death at infection sites in a short time (<24 h) to restrict pathogen growth, while plants with field resistance might take up to several days to response. Fourth, the number (23.4) of TDFs per primer combination was lower comparing to a previous study of 48.8 TDFs per primer combination in the tomato-*Cladosporium fulvum* pathosystem [25], which could also contribute to the less genes identified in this study. However, the ratio of differentially expressed TDFs (1.2%) in this study was close to 1% in Gabriëls et al. [25]. Therefore, cDNA-AFLP technique could be used to identify genes differently expressed in tomato plants during the infection of bacterial spot pathogen.

The genetics of field resistance to bacterial spot race T3 in tomato line PI 114490 is complicated. At least five QTLs on chromosomes 1, 3, 8, 11, and 12 with small effect (6.5–11.7%) have been reported to date [14], [15], suggesting that the high level of field resistance in PI 114490 requires interaction of some of these QTLs [14]. In the present study, 31 of 71 genes were on the five chromosomes having QTLs and 12 genes were at the QTL regions. Particularly, the TDF785 (Solyc11g051200.1.1, Cytochrome P450 like_TBP) locating on the QTL region of chromosome 11 was exclusively up-regulated in PI 114490, indicating that this TDF might be associated with the QTL. In addition, although most TDFs showed up-regulated in both PI 114490 and OH 88119, the expression patterns were different. For example, the up-regulation of TDF721 showed a reduction trend at 3, 4, and 5 DPI in PI 114490, but an increase trend in OH 88119 (Fig. 5). Therefore, the regulatory mechanism could also be complicated.

Three pathways, Microbe/Pathogen-Associated Molecular Patterns recognition, effector recognition, and phytohormone pathways, have been proposed for plant immune response. Each pathway involves in interactions among many genes. Phytohormones including salicylic acid (SA), gaseous ethylene (ET), and jasmonic acid (JA) can regulate local and systemic resistance to invasive pathogens [40]. The plant hormone ET is an important component of defense signaling [41], [42]. Genes encoding AP2-like ethylene-responsive transcript factor (AP2/ERF) and 1-aminocyclopropane-1-carboxylate oxidase (ACC) play an important role in response to mechanical damage and symptom development by pathogen attack [35], [43], [44]. Tomato ethylene-responsive transcript factor (ERF) Pti4, Pti5 and Pti6 directly interact with *Pto* resistance gene [42] and play a role in activation of pathogenesis-related genes, resulting in enhanced defense against certain bacterial and fungal pathogens [45]–[47]. Based on the annotation, the TDF87 identified in this study is a member of the ERF protein family. It showed different levels of up-regulation in both resistant tomato line PI 114490 and susceptible line OH 88119 during the *X. perforans* race T3 infection, suggesting that it might contribute to the defense against the pathogen. In addition to its contributing to resistance in some plant-pathogen interactions, ethylene is also involved in chlorosis symptom development. The ethylene -insensitive Arabidopsis and soybean plants showed less chlorosis symptoms than wild-type plants [48], [49]. The TDF721, a member of ACC family, was up-regulated during the pathogen infection in tomato lines PI 114490 and OH 88119. However, the resistant line PI 114490 showed less disease symptom on leaves than the susceptible line OH 88119, which might due to the differential up-regulation of TDF721 in two tomato lines.

Many plant pathogens have evolved virulence strategies to regulate JA signaling pathway in their hosts to facilitate infection and production of disease symptoms [50], [51]. We here identified four up-regulated genes (TDF281, TDF711, TDF741, and TDF744) that might contribute to the regulation of JA biosynthesis during tomato-*X. perforans* interactions. TDF281 and TDF711 were homologous to different Cytochrome P450 (Table 1). They were induced in both PI 114490 and OH 88119 but the expressions were higher in OH 88119 than in PI 114490 at 4 and 5 DPI (Fig. 5). Blast search revealed that both genes contain allene oxide synthase activity, which is the key enzyme in the JA biosynthesis pathway [52], [53]. TDF741 and TDF744 were annotated as Jasmonate ZIM domain 2 (JAZ2) and Jasmonate ZIM-domain protein 3 (JAZ3), respectively (Table 1). The differentially expressed JAZ genes were also observed in bean-*Xanthomonas axonopodis* pv. *phaseoli* interaction [54] and *Arabidopsis* -*Pseudomonas syringae* interactions [51]. Characterization of mutants encoding truncated JAZ proteins and RNAi line have demonstrated that some JAZ proteins are both negative regulators of JA signaling and disease symptom development [51], [55], [56]. Therefore, it is very likely that these two TDFs might play important roles in altering JA signaling to impact on symptom development in tomato-*X. perforans* race T3 interaction.

In summary, response to *X. perforans* in tomato plants involves a complicated network regulated by a large number of defense/pathogenesis-related genes. Using the cDNA-AFLP technique, a total of 79 TDFs representing 71 genes with different expression patterns during the pathogen infection were identified in this study. Differential expression of several JA- and ET-related genes during the process of the pathogen infection indicates that co-activation of JA and ET signaling pathways may play key roles in response to *X. perforans* race T3 in tomato plants.
